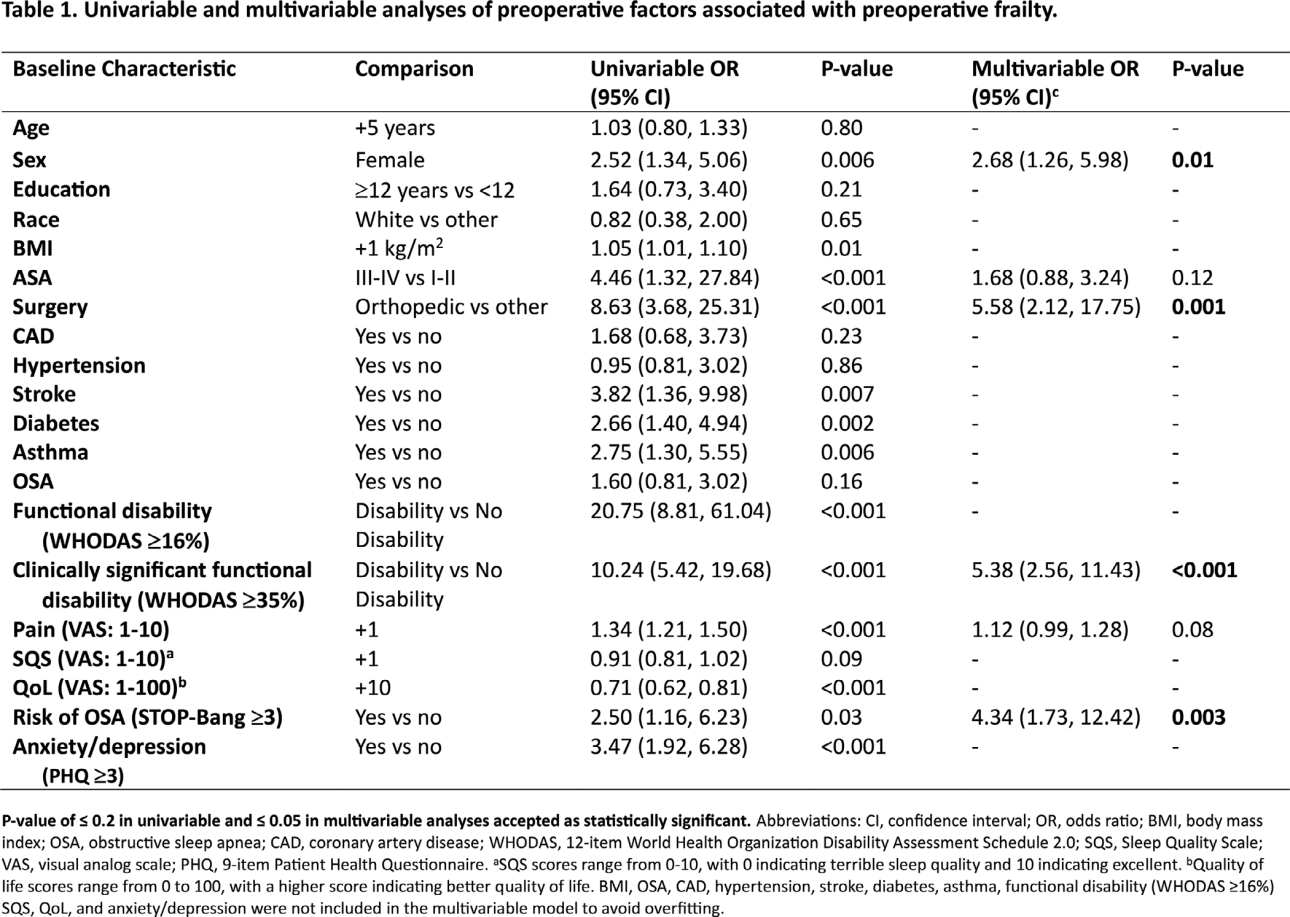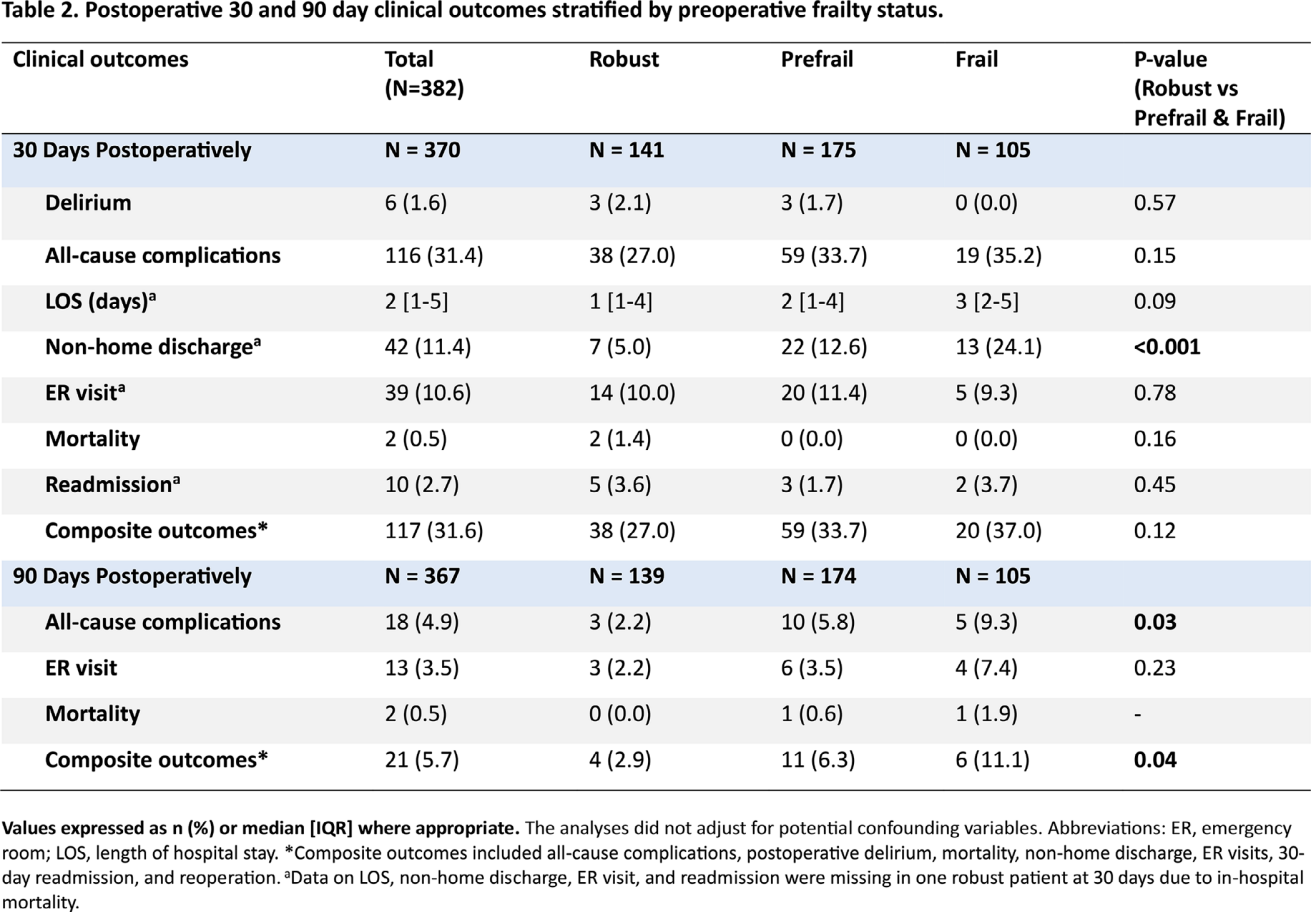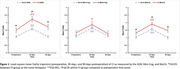# Assessing the association between cognitive impairment and perioperative trajectory of frailty in older adults undergoing anesthesia and surgery: a multicentred cohort study

**DOI:** 10.1002/alz70857_097144

**Published:** 2025-12-24

**Authors:** Eric Ka J Cheuk, Ellene Yan, Yasmin Alhamdah, Sinead Campbell, David He, Jean Wong, Frances Chung

**Affiliations:** ^1^ Temerty Faculty of Medicine, University of Toronto, Toronto, ON, Canada; ^2^ Toronto Western Hospital, University Health Network, Toronto, ON, Canada; ^3^ Women's College Hospital, Toronto, ON, Canada; ^4^ Department of Anesthesia and Pain Management, Mount Sinai Hospital, Toronto, ON, Canada; ^5^ Mount Sinai Hospital, Toronto, ON, Canada

## Abstract

**Background:**

Cognitive impairment (CI) and frailty can lead to increased vulnerability and reduced capacity to cope with anesthesia and surgery. Understanding the trajectory of frailty in those with versus without CI can help enhance perioperative care. This study aimed to (1) compare the prevalence and trajectory of frailty between patients with and without CI following surgery, (2) investigate patient‐centered variables associated with preoperative frailty, and (3) examine clinical outcomes by baseline frailty.

**Method:**

Patients ≥65 years old undergoing elective non‐cardiac surgery provided informed consent following ethics board approval. Preoperative CI was assessed using the Ascertain Dementia Eight‐item Questionnaire (AD8) (≥2), Mini‐Cog (≤2), and Montreal Cognitive Assessment (MoCA) (≤25). Frailty was measured by the 5‐item FRAIL Questionnaire (prefrail: 1‐2; frail: ≥3) preoperatively and at 30 and 90 days. Uni‐ and multivariable logistic regression was performed to determine patient‐centered variables associated with preoperative frailty. Clinical outcomes at 30 and 90 days were collected from electronic medical records.

**Result:**

Of 381 participants, 13.6%, 12.6%, and 34.1% screened positive for CI on the AD8, Mini‐Cog, and MoCA, respectively. Among the same assessments, 29.1%, 12.7%, and 40.0% CI patients were also frail preoperatively. In contrast to the Mini‐Cog, patients with CI on the AD8 or MoCA were significantly frailer than those without CI at all timepoints (Figure 1). Regardless of cognitive assessment tool, frailty improved across all timepoints for both CI and no‐CI patients, with both groups exhibiting a similar rate of improvement (Figure 1). In the multivariable analysis, females (3‐fold), orthopedic surgery (6‐fold), significant preoperative functional disability (5‐fold), and high risk of obstructive sleep apnea (4‐fold) were associated with higher odds of preoperative frailty, after adjusting for American Society of Anesthesiologists (ASA) class and pain (Table 1). Both frail and prefrail patients had significantly higher incidences of non‐home discharge at 30 days, all‐cause complications at 90 days, and composite adverse outcomes at 90 days (Table 2).

**Conclusion:**

Our findings demonstrated greater prevalence of frailty in CI patients, with frail and prefrail patients experiencing poorer postsurgical outcomes. Early detection of frailty and CI can inform tailored perioperative management strategies to enhance recovery, optimize patient‐centered care and clinical outcomes.